# Use of gene expression and whole-genome sequence information to improve the accuracy of genomic prediction for carcass traits in Hanwoo cattle

**DOI:** 10.1186/s12711-020-00574-2

**Published:** 2020-09-29

**Authors:** Sara de las Heras-Saldana, Bryan Irvine Lopez, Nasir Moghaddar, Woncheoul Park, Jong-eun Park, Ki Y. Chung, Dajeong Lim, Seung H. Lee, Donghyun Shin, Julius H. J. van der Werf

**Affiliations:** 1 School of Environmental and Rural Science, University of New England, Armidale, NSW 2351 Australia; 2 Animal Genomics and Bioinformatics Division, National Institute of Animal Science, Rural Development Administration, Wanju, 55365 Republic of Korea; 3 Department of Beef Science, Korea National College of Agriculture and Fisheries, Jeonju, Republic of Korea; 4 Division of Animal and Dairy Science, Chungnam National University, Deajeon, 34148 Republic of Korea; 5 The Animal Molecular Genetics and Breeding Centre, Jeonbuk National University, Jeonju, 54896 Republic of Korea

## Abstract

**Background:**

In this study, we assessed the accuracy of genomic prediction for carcass weight (CWT), marbling score (MS), eye muscle area (EMA) and back fat thickness (BFT) in Hanwoo cattle when using genomic best linear unbiased prediction (GBLUP), weighted GBLUP (wGBLUP), and a BayesR model. For these models, we investigated the potential gain from using pre-selected single nucleotide polymorphisms (SNPs) from a genome-wide association study (GWAS) on imputed sequence data and from gene expression information. We used data on 13,717 animals with carcass phenotypes and imputed sequence genotypes that were split in an independent GWAS discovery set of varying size and a remaining set for validation of prediction. Expression data were used from a Hanwoo gene expression experiment based on 45 animals.

**Results:**

Using a larger number of animals in the reference set increased the accuracy of genomic prediction whereas a larger independent GWAS discovery dataset improved identification of predictive SNPs. Using pre-selected SNPs from GWAS in GBLUP improved accuracy of prediction by 0.02 for EMA and up to 0.05 for BFT, CWT, and MS, compared to a 50 k standard SNP array that gave accuracies of 0.50, 0.47, 0.58, and 0.47, respectively. Accuracy of prediction of BFT and CWT increased when BayesR was applied with the 50 k SNP array (0.02 and 0.03, respectively) and was further improved by combining the 50 k array with the top-SNPs (0.06 and 0.04, respectively). By contrast, using BayesR resulted in limited improvement for EMA and MS. wGBLUP did not improve accuracy but increased prediction bias. Based on the RNA-seq experiment, we identified informative expression quantitative trait loci, which, when used in GBLUP, improved the accuracy of prediction slightly, i.e. between 0.01 and 0.02. SNPs that were located in genes, the expression of which was associated with differences in trait phenotype, did not contribute to a higher prediction accuracy.

**Conclusions:**

Our results show that, in Hanwoo beef cattle, when SNPs are pre-selected from GWAS on imputed sequence data, the accuracy of prediction improves only slightly whereas the contribution of SNPs that are selected based on gene expression is not significant. The benefit of statistical models to prioritize selected SNPs for estimating genomic breeding values is trait-specific and depends on the genetic architecture of each trait.

## Background

Korean beef production is mainly based on the Hanwoo breed and production efficiency has improved significantly since the beginning of 1983 when performance and progeny testing was introduced in the Hanwoo breeding program [1]. In particular, marbling score has improved considerably due to combining improved feeding strategies (feeding more concentrates and for extended periods in the feedlot) and breeding strategies such as sire selection and artificial insemination. The high value of Hanwoo meat has motivated further genetic improvement of economically important carcass traits such as marbling score (MS), eye muscle area (EMA), back fat thickness (BFT), and carcass weight (CWT). In recent years, with the availability of new genomic and bioinformatics tools, approaches that use genotype information for the selection of Hanwoo cattle are being introduced because it can increase rates of genetic gain due to increased selection accuracy and decreased generation interval for the selection of breeding bulls [1]. Merban et al. [2] investigated the accuracy of genomic prediction for carcass traits in Hanwoo cattle by evaluating different models. They found that the accuracy of prediction for CWT increased when using BayesC compared with genomic best linear unbiased prediction (GBLUP) and Bayesian LASSO, but that there was no difference in accuracy between the three methods for BFT, EMA, and MS [2]. However, in this study sample size (1183 animals) and single nucleotide polymorphisms (SNP) density (34,000 SNPs) were considered to be limiting factors. The availability of sequence information and an increased number of genotyped animals provide scope for improving accuracy of prediction. Imputed full sequence information applied in standard GBLUP procedures does not necessarily improve the accuracy of prediction or explain more genetic variation of the traits compared with the use of 50 k or high-density (HD) SNP arrays [3–5]. A more efficient use of sequence information can be achieved by selecting SNPs based on their effect on phenotypic differences or on knowledge of the biology of the traits, i.e. which genes are likely involved, and such information could be obtained from association studies or from other sources of information such as gene expression experiments.

In dairy cattle [4, 6, 7] and sheep [8], pre-selection of SNPs from whole-genome sequence data based on either candidate genes or genome-wide association studies (GWAS) have resulted in small to moderate improvements in accuracy of prediction. GWAS in Hanwoo have provided information about the genetic architecture of carcass traits [9–14] and sensory traits [15]. In general, these studies suggest that carcass traits are polygenic since numerous quantitative trait loci (QTL) regions were detected, each with a small effect [14, 16]. However, only a few QTL overlap between these studies in Hanwoo, which is likely due to the relatively small datasets used to identify QTL regions and the low to moderate marker density. QTL detection and identification of predictive SNPs can be improved with larger datasets and high-density genotype information, e.g. at the sequence level.

The use of more biological information (gene expression, methylation, protein, and metabolite data, among others) has been recommended to identify genomic features that are enriched for causal variants in complex traits. In dairy cattle, the addition of predictive SNPs for milk traits increased the accuracy by 16% in the Australian Red breed. In particular, prioritization of SNPs in coding and regulatory regions in a BayesRC model increased the accuracy of prediction for milk traits compared to using only high-density genotypes [17]. In another study, using information from the best-performing gene ontology term in a genomic feature BLUP (GFBLUP) model increased, on average, accuracy of prediction for milk traits (milk, fat, protein, and mastitis) by 0.02 points [18]. Xiang et al. [19] found that the identification of informative SNPs (high-ranking variants) based on a functional and evolutionary trait heritability (FAETH) score from multiple sources of information resulted in higher heritability and increased accuracy of prediction than the use of low ranking variants in Holstein and Danish Red cattle [19].

Information on genes that are involved in marbling phenotypes in Hanwoo cattle has been generated from a time-series gene expression experiment using quantitative PCR (qPCR) [20–23] and whole-transcriptome analysis and has led to the identification of eQTL and SNPs located in genes that are associated with marbling [24]. The aim of our study was to assess the prediction accuracy of GBLUP, wGBLUP, and Bayesian models for carcass traits (CWT, MS, EMA, and BFT) and the potential gain from using pre-selected SNPs from a GWAS on imputed sequence data and from gene expression information in Hanwoo cattle.

## Methods

### Animals and phenotypes

All the procedures described in this paper were in accordance with the protocols accepted by the Animal Care and Use Committee of the National Institute of Animal Science in the Republic of Korea (Approval No. 2018–293). The genotypes and phenotypes from 13,717 Hanwoo cattle were collected by the Animal Genomics and Bioinformatics Division of the National Institute of Animal Science (RDA) between 2000 and 2016 in commercial slaughterhouses in South Korea. Four carcass traits were analyzed; cold carcass weight (CWT, kg) was recorded for each animal after 24 h of chilling whereas marbling score (MS), eye muscle area (EMA, cm^2^), and subcutaneous back fat thickness (BFT, mm) were measured on the *longissimus dorsi* muscle between the 13th rib and the 1st lumbar vertebra. Marbling scores were assigned based on the Korean Beef Marbling visual standard category system (KAPE, 2012), which accounts for the percentage of intramuscular fat (IMF) observed from 1 (no IMF) to 9 (19% or more IMF).

### RNA-seq analysis

The transcriptomic analysis was performed on 45 Hanwoo steers, which were fed high (23 steers) and low (22 steers) energy diets. *Longissimus dorsi* muscle was sampled by biopsy between the 10th and 13th cervical vertebra at 7, 8, 12, 18, and 24 months of age, and collected after slaughter at 30 months of age. For the biopsies, each steer was restrained in a hydraulic squeezed chute, hair was removed from the biopsy site, and local anesthetic (lidocaine HCl; 20 mg/mL; 8 mL per biopsy site) was administered. The biopsy site was cleaned with 70% ethanol on sterile surgical gauze and a 1 to 3 cm skin incision was made with a sterile scalpel. Tissue (1 to 2 g) was collected from the muscle using a sterile Bergstrom biopsy needle (5.3 mm diameter) and was preserved in liquid nitrogen and stored at − 80 °C. After closing the incision site with veterinary tissue glue, the area was covered with a spray-on aluminum bandage. All steers were monitored for swelling 24 and 48 h after biopsy. Samples were obtained on alternate sides of the animal depending on the location of the previous sampling, and the third and fourth biopsies were sampled 5 cm away from the location of the previous biopsy.

RNA was extracted from the isolated tissues using the TRIzol reagent (Invitrogen, Life Technology, Carlsbad, USA) following the manufacturer’s recommendations. The quality of the RNA was evaluated on a Bioanalyzer 2100 with RNA 6000 Nano Labchips (Agilent Technologies Ireland, Dublin). The high-quality RNA samples with an average RNA integrity value higher than 7 were used to produce indexed shotgun paired-end (PE) libraries with on average 500 bp inserts generated using a TruSeq Nano DNA Library Prep Kit (Illumina, USA) following the standard Illumina sample-preparation protocol. The resulting libraries were sequenced on an Illumina HiSeq 2500 sequencer (2 × 101 bp paired-end sequences).

The quality of the reads was assessed with the FastQC tool [25], low-quality bases (Phred < 33) and adapters were removed using Trimmomatic v0.32 [26]. We mapped the resulting reads to the *Bos taurus* reference genome (version UMD 3.1) using the HISAT2 v2.1.0 [27] software and the R function *featureCount* from the package Rsubread v1.34.7 to obtain the gene counts [28].

### Genotypes and imputation

The 50 k SNP genotypes and sequences were obtained from an Illumina platform and SNP locations were derived using the *Bos taurus* reference genome version UMD 3.1. Quality control thresholds were set to filter out SNPs with a minor allele frequency lower than 0.01, that deviated from Hardy–Weinberg disequilibrium (*p* < 10^−6^) and to exclude individuals with more than 5% missing genotypes. A subset of 4566 animals (4452 of those with recorded phenotypes for carcass traits) with previously imputed whole-sequence genotypes, based on 203 fully sequenced Hanwoo animals, were used as a reference for imputing genotypes of the remaining 10,215 samples to high-density and whole-sequence genotype data. Genotypes were phased with the software Eagle v2.4.1 [29] and we used Minimac3 [30] to impute the 50 k genotypes (58,991 SNPs) of 10,215 animals to high-density (HD; 543,263 SNPs) and finally up to whole-sequence (10,723,697SNPs). The final imputed sequence data (that included only SNPs with a Minimac3 R^2^ > 0.4) consisted of 10,723,697 SNPs with an average imputation accuracy (R^2^) of 0.99 from Minimac3.

### Selection of the discovery datasets for GWAS

In order to select the statistically most significant SNPs to improve accuracy of genomic prediction, an association study is required. For this purpose, a discovery set needs to be extracted from the data that is independent of the set used for training and validating accuracy of prediction [17]. A larger discovery set allows a more accurate association study but leaves a smaller cross-validation set to train and test predictions. We evaluated the impact of using different numbers of animals in the discovery set on the identification of SNPs associated with carcass traits and the accuracy of prediction in Hanwoo cattle. To achieve this, four discovery datasets (1000, 2000, 3000, and 4000 animals) were extracted from the complete dataset of 13,717 animals. We selected animals for the discovery set such that the genetic diversity in each set was high by selecting the set (out of one hundred random samples) that had the smallest average co-ancestry according to the following criterion:

$$\sum\limits_{i\; = 1}^{n} {\frac{{{\mathbf{x}}'{\mathbf{Gx}}}}{2}} ,$$

where ${\mathbf{x}}$ is an indicator vector of the selected animals (1 or 0 if selected, or not), and ${\mathbf{G}}$ is the genomic relationship matrix for all the animals. The genomic relationship matrix was calculated with 50 k SNP genotypes from all the animals using the Plink v1.90b4 software [31].

### Selection of cross-validation datasets

After selecting a discovery set, the remaining animals were used for a tenfold cross-validation (CV) to evaluate the accuracy and bias of genomic prediction. Depending on the number of animals used in the discovery dataset, between 9717 and 12,717 animals were available for CV. The effect of forming the 10 subsets for CV, randomly or by k-means clustering, was assessed by comparing the impact of these strategies on accuracy of prediction. Note that there were no sire-son relationships in the data, since all phenotypes were on commercially slaughter animals. However, sib relationships might exist, but no pedigree was available. Therefore, samples were grouped based on a k-mean strategy using the *kmeans* R function from the stats package, which basically uses as input the principal components matrix of the genomic relationships (from the 50 k SNP chip) to cluster the samples with a smaller within-cluster variation. The number of clusters was set to 100, whereas the number of random starting partitions was 50. Based on the assigned clusters, the samples were then split into 10 groups with a similar number of animals. It is expected that the k-means clustering results in a lower degree of genetic relatedness between the validation and training sets, leading to a lower accuracy of genomic prediction but to a greater benefit from using selected SNPs in the prediction model.

To compare the different prediction methods with the selected SNPs, we decided to use a k-means strategy for selecting the validation and reference sets. This strategy decreased the relatedness between training and validation sets, which likely resulted in a greater benefit from using pre-selected SNPs from prediction [8] and therefore allowed a better comparison of methods. Moreover, in breeding programs, often the animals for which breeding values need to be predicted are relatively less related to the nucleus, and such a situation can be simulated with the k-means strategy.

### Statistical analysis

All the traits were adjusted for fixed effects based on a univariate analysis in ASReml v4.1 [32]. The evaluated fixed effects were birth-year (15 levels: 2000 to 2016), birth-month (12 levels), age at slaughter (from 17 to 173 months), slaughter-year (9 levels: 2008 to 2018), slaughter-month (12 levels); slaughter-place (53 levels), herds of origin (324 levels), and sex (2 levels).

The fixed effects that were significant for all traits were herd of origin, birth-year, birth-month, slaughter-year, slaughter-month, slaughter-place, age, and sex. The interaction effect of herd $\times$ birth-year was fitted in a final model for CWT without fitting the main effects whereas a herd $\times$ birth-year $\times$ birth-month effect was fitted for MS and BFT. The adjusted phenotypes were obtained as the residuals from the fitted model for each carcass trait.

### Pre-selection of SNPs and GWAS

A genome-wide association analysis (GWAS) was applied to each of the carcass traits based on each of the individual variants from the imputed sequence data, fitting a univariate linear mixed model using the GCTA v1.26.0 software [33]:

1 $${\mathbf{y}} = {\mathbf{1}}\mu + {\mathbf{x}}_{i} \alpha_{i} + {\mathbf{Za}} + {\mathbf{e}},$$

where ${\mathbf{y}}$ is the vector of ${\text{N}}$ adjusted phenotypic values (for MS, BFT, EMA, or CWT), $1$ is a vector with ${\text{N}}$ ones, $\mu$ is the intercept, ${\mathbf{x}}_{i}$ is a vector of the genotypes at a single SNP $i$, $\alpha_{i}$ is the regression coefficient for the allele substitution effect of SNP $i$, ${\mathbf{Z}}$ is an incidence matrix for animals, ${\mathbf{a}}$ is vector of the random additive genetic effects of animals, and ${\mathbf{e}}$ is a vector of random residual effects; ${\text{var}}\left( {\mathbf{a}} \right) = {\mathbf{G}}\sigma_{\text{a}}^{2}$ where ${\mathbf{G}}$ is the genomic relationship matrix based on the imputed sequence genotypes and ${\text{var}}\left( {\mathbf{e}} \right) = {\mathbf{I}}\sigma_{\text{e}}^{2}$.

For each trait, significant SNPs were selected based on seven thresholds for the − log_10_(*p* value) (1.5, 2, 2.5, 3, 3.5, 4, or 5, respectively). For each list of top SNPs, the software Plink was used to prune SNPs that were in high linkage disequilibrium (LD) with other SNPs in the region. SNPs with an r^2^ value higher or equal to 0.95 with the most significant SNP in a window of 5000 SNPs were removed, and the process was repeated after each window shift of 100 SNPs.

The proportion of genetic variance was calculated for significant SNPs as:

$$\frac{{2p_{i} (1 - p_{i} )\alpha_{i}^{2} }}{{\sigma_{a}^{2} }} \times 100,$$

where $p_{i}$ is the allele frequency of the reference allele of SNP $i$, $\sigma_{a}^{2}$ is the additive genetic variance of the trait, and $\alpha$ is the estimated additive effect of SNP $i$.

### Pre-selection of SNPs and gene expression

Two strategies were followed to preselect SNPs from expression studies: (1) SNPs in genes the expression of which was associated with the traits, and (2) SNPs from expression QTL (eQTL). To evaluate the association of each gene expression with each of the traits (MS, CWT, EMA, and BFT) a linear regression of gene count on phenotype was used by fitting the feeding treatment and sires as fixed effect. We selected a significance threshold *p*-value < 0.0032 (− log_10_ *p*-value = 2.5) to identify genes that were significantly associated (GSA) with a trait. For each GSA, relevant SNPs were identified in the imputed sequence data from the promoter region (300 bp before the transcription start site) to the end of the gene.

For the identification of eQTL, an association analysis of each SNP with each gene expression (13,572 genes) was performed with the R package MatrixEQTL v2.2 [34] using all imputed sequences SNPs of 45 animals and the gene expression at each of the five ages. The effect of the genotype on expression was assumed to be additive linear (modelLINEAR), by fitting the feeding treatments and sires as fixed effects in the model. The local (*cis*-) and distal (*trans*-) eQTL were identified using a significance threshold of *p*-value < 0. 0032.

The lists of eQTL were pruned in the same way as the GWAS SNPs by using Plink to remove the SNPs in high LD (r^2^ ≥ 0.95) with the most significant SNPs in a window of 5000 SNPs and repeating the process after shifting the window by 100 SNPs.

### Genomic prediction

We used the MTG2 v9.09 software [35] to estimate variance components with restricted maximum likelihood (REML) and to calculate the genomic breeding values (GBV) using all the data that were not included in the GWAS discovery dataset. The accuracy of prediction was assessed for two GBLUP models:

2 $${\mathbf{y}} = {\mathbf{1}}\mu + {\mathbf{Zg}}_{1} + {\mathbf{e}}$$

3 $${\mathbf{y}} = {\mathbf{1}}\mu + {\mathbf{Zg}}_{1} + {\mathbf{Zg}}_{2} + {\mathbf{e}}$$

where ${\mathbf{y}}$ is the vector of ${\text{N}}$ adjusted carcass trait phenotypes (MS, CWT, EMA, and BFT), **1** is a vector with ${\text{N}}$ ones, $\mu$ is intercept, ${\mathbf{Z}}$ is the design matrix to assign ${\mathbf{y}}$ to ${\mathbf{g}}$ (1 or 2), ${\mathbf{g}}_{1}$ and ${\mathbf{g}}_{2}$ are the additive genetic effects of individuals with ${\text{var}}\left( {{\mathbf{g}}_{i} } \right) = {\mathbf{G}}_{i} \sigma_{a}^{2}$, and ${\mathbf{e}}$ is the vector of residual effects. Standard 50 k SNP chip (40,933 SNPs) genotypes were used to calculate the ${\mathbf{G}}_{1}$ used for ${\mathbf{g}}_{1}$ (Model 3), while the pre-selected SNPs (top SNPs with *p* < 0.0032 from GWAS or SNPs from GSA and eQTL) were used in a second ${\mathbf{G}}_{2}$ as a covariance matrix for ${\mathbf{g}}_{2}$ in Model 3, and in this case the overlapping top SNPs were removed from ${\mathbf{G}}_{1}$ and the resulting matrix is referred to as **G**_50adj_. The sum of ${\mathbf{g}}_{1}$ and ${\mathbf{g}}_{2}$ is the estimated breeding value (GBV) in Model 3.

A weighted-GBLUP (wGBLUP) procedure was implemented for the reference-validation dataset of 10,717 animals. This procedure is described in detail in [36]. Briefly, in the wGBLUP method, the ${\mathbf{G}}$ matrix is constructed by using the weight of each SNP and this weight is based on the estimated effect of the SNP on the trait, as derived from back-solving for SNP effects from the estimated genomic breeding values [36, 37], and recalculating these through five iterations. Following VanRaden [38], SNP weights were calculated as ${\text{d}}_{{{\text{i}}\left( {{\text{t}} + 1} \right)}} = 1.25^{{\frac{{\left| {{\hat{\mathbf{\alpha }}}_{i} } \right|}}{{sd\left( {{\hat{\mathbf{\alpha }}}} \right)}} - 2}}$, where $\alpha$ are estimated SNP effects in a previous iteration round.

A Bayesian mixture model was fitted by applying a Markov chain Monte Carlo (MCMC) method with 50,000 iterations (20,000 iterations were burn-in) to estimate parameters using the BayesR method as implemented by [39]. The model uses a mixture of four distributions for the effects of SNPs with variances in each distribution being 0, 0.0001, 0.001, and 0.01 of $\sigma_{g}^{2}$, respectively, $\sigma_{g}^{2}$ being estimated from the data. The BayesR model for prediction was:

4 $$\hat{y}\; = \;\mu + \sum\limits_{{j \in \beta_{j} > 0}} {w_{ij} \hat{\beta }_{J} } ,$$

where $\hat{\beta }_{j}$ is the estimated effect of SNP $j$, $w_{ij} = \frac{{\left( {x_{ij} - 2p_{j} } \right)}}{{\sqrt {2p_{j} \left( {1 - p_{j} } \right)} }}$ and $x_{ij}$ is the number of copies of the reference allele in the reference genome (0, 1, 2) at SNP $j$ for individual $i$ and $p_{j}$ is the frequency of the reference allele.

Eight strategies were followed to compare the use of pre-selected SNPs in the estimation of breeding values for the carcass traits: (1) GBLUP-50 k: Model 2 with only the standard 50 k array to calculate the genomic relationship matrix (${\mathbf{G}}_{1}$); (2) GBLUP-GWAS: using Model 3 with the top SNPs derived from GWAS to construct ${\mathbf{G}}_{2}$ along with the adjusted 50 k ${\mathbf{G}}$ (${\mathbf{G}}_{{50{\text{adj}}}}$) as ${\mathbf{G}}_{1}$; (3) GBLUP-eQTL: using Model 3 with the selected eQTL to form the ${\mathbf{G}}_{2}$ together with the adjusted 50 k ${\mathbf{G}}$ (${\mathbf{G}}_{{50{\text{adj}}}}$) as ${\mathbf{G}}_{1}$; (4) GBLUP_GSA: using Model 3 with SNPs identified in GSA and differentially expressed (DE) genes to form the ${\mathbf{G}}_{2}$ in combination with the 50 k ${\mathbf{G}}$ (${\mathbf{G}}_{{50{\text{adj}}}}$); (5) wGBLUP-50 k: fitting a weighted-GBLUP procedure applied to Model 2 using the standard 50 k SNP array to form ${\mathbf{G}}_{1}$; (6) wGBLUP-GWAS: using the standard 50 k and top SNPs in one genomic relationship matrix as ${\mathbf{G}}_{1}$ in Model 2 and applying a wGBLUP procedure; (7) BayesR-50 k: performing a BayesR analysis with the standard 50 k SNP array; and (8) BayesR-GWAS: using a BayesR model with genomic information that includes the standard 50 k SNP array as well as the top SNPs.

### Accuracy of prediction

The accuracy of prediction was assessed as the Pearson’s correlation coefficient (r) between the adjusted phenotype of the animals in the validation dataset and their GBV divided by the square root of the heritability for each trait. The empirical standard error (SE) was calculated by dividing the standard deviation of the 10 calculated accuracies from the tenfold CV by the square root of 10. The bias of the genomic prediction was calculated as the deviation from unity of the regression coefficient (*b*) of the phenotype on the GBV.

## Results

The basic statistics and the estimated variance components and heritability for EMA, BFT, CWT, and MS are in Table 1 for Model 1 and Table 2 for Model 2. The heritabilities ranged from 0.24 to 0.27. The sum of the two additive genetic components of the variance in Model 2 tended to be somewhat smaller than the additive genetic component in Model 1.

**Table 1:** Descriptive statistics and variance components (standard error in brackets) estimated for the carcass traits in Hanwoo cattle (n = 13,717)

	BFT	EMA	CWT	MS
*h*^*2*^	0.24 (0.01)	0.24 (0.01)	0.25 (0.01)	0.27 (0.01)
$\sigma_{a}^{2}$	4.8 (0.33)	27.6 (1.80)	571.4 (36.44)	0.66 (0.04)
$\sigma_{p}^{2}$	20.1 (0.28)	113.5 (1.56)	2272.5 (31.30)	2.44 (0.03)
$\sigma_{e}^{2}$	15.3 (0.26)	85.9 (1.47)	1701.1 (29.18)	1.77 (0.03)
Min	1	22	152	1
Max	57	156	692	9
Mean	13.42	92.61	425.50	5.68
SD	5.23	12.56	59.84	1.98

*h*^*2*^: estimated heritability; $\sigma_{a}^{2}$: additive genetic variance; $\sigma_{p}^{2}$: phenotypic variance; $\sigma_{e}^{2}$: residual variance; SD: standard deviation

*Max* maximum value, *Min* minimum value of adjusted phenotypes for *BFT* back fat thickness, *EMA* eye muscle area, *CWT* carcass weight and *MS* marbling score

**Table 2:** Variance components (standard error in brackets) for two ${\mathbf{G}}$ matrices estimated for the carcass traits in Hanwoo cattle (n = 10,717)

	*h*^*2*^	$\varvec{\sigma}_{\varvec{a}}^{2}$	$\varvec{\sigma}_{\varvec{e}}^{2}$
BFT			
${\mathbf{G}}_{1}$	0.15 (0.02)	2.85 (0.34)	15.15 (0.29)
${\mathbf{G}}_{2}$	0.07 (0.01)	1.30 (0.20)	
EMA			
${\mathbf{G}}_{1}$	0.16 (0.02)	18.42 (2.00)	88.13 (1.66)
${\mathbf{G}}_{2}$	0.05 (0.01)	5.73 (1.21)	
CWT			
${\mathbf{G}}_{1}$	0.12 (0.02)	251.95 (34.72)	173.76 (32.08)
${\mathbf{G}}_{2}$	0.08 (0.01)	177.46 (25.15)	
MS			
${\mathbf{G}}_{1}$	0.18 (0.02)	0.43 (0.04)	1.83 (0.04)
${\mathbf{G}}_{2}$	0.06 (0.01)	0.14 (0.03)	

*h*^*2*^: estimated heritability; $\sigma_{a}^{2}$: additive genetic variance; $\sigma_{e}^{2}$: residual variance; ${\mathbf{G}}_{1}$: adjusted ${\mathbf{G}}$ matrix with 50 k SNP array (top SNPs removed); ${\mathbf{G}}_{2}$: ${\mathbf{G}}$ matrix with the top-SNPs from the 3000 discovery dataset

*BFT* back fat thickness, *EMA* eye muscle area, *CWT* carcass weight, *MS* marbling score

### Genomic prediction of carcass traits

#### Comparison of k-means or random selection strategies for cross-validation

The accuracy of genomic selection and the added value of using top SNPs likely depends on the genetic diversity of the population and the degree of relatedness between reference set and validation set. Two sampling strategies were compared for selecting the validation and reference sets: k-means clustering and random sampling. In total, 13,717 Hanwoo cattle were used for the comparison of accuracy of prediction for the carcass traits based on the standard 50 k panel in a tenfold CV.

**Fig. 1:**
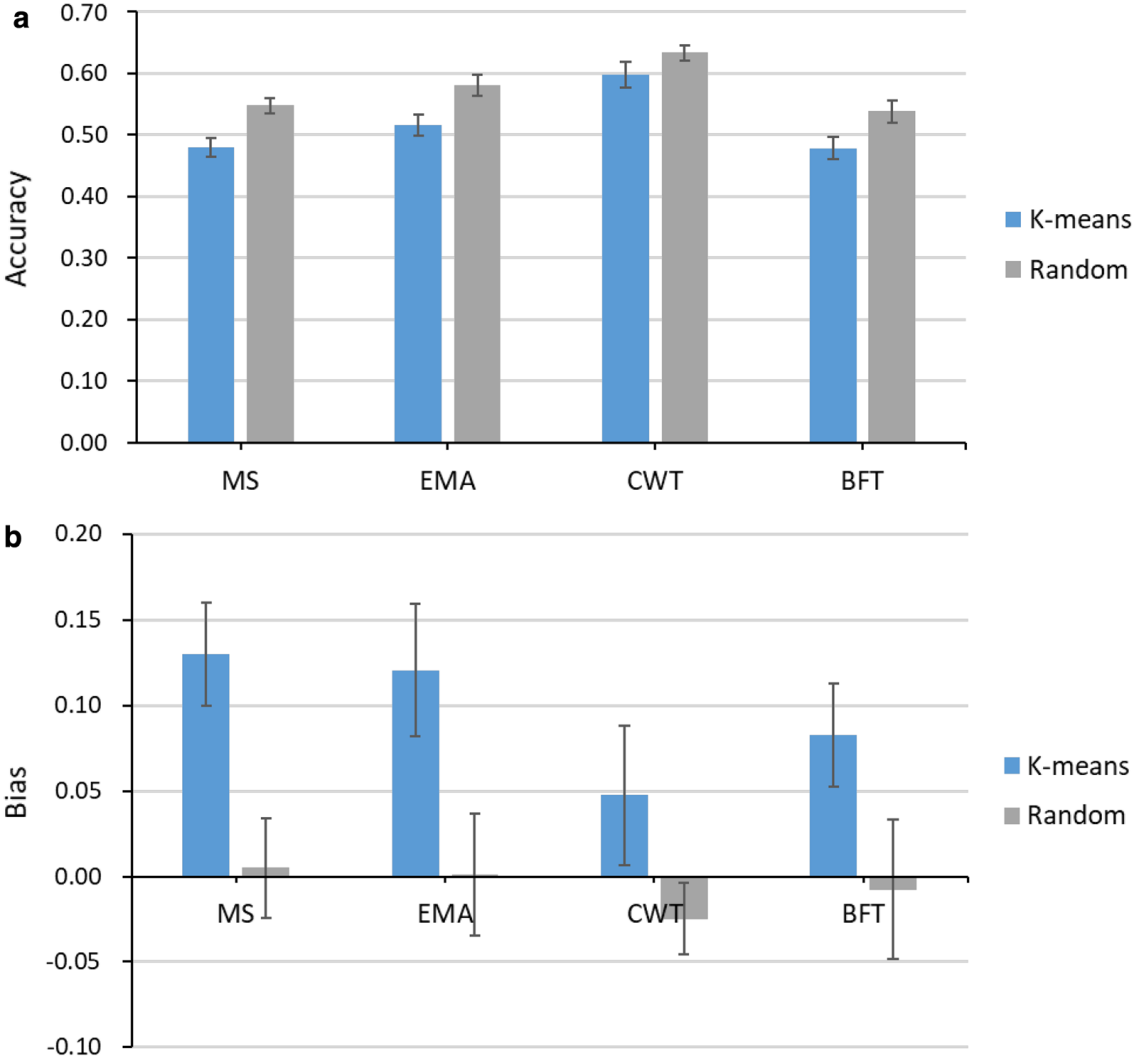
Accuracy (**a**) and bias (**b**) of genomic prediction of breeding value for the carcass traits marbling score (MS), eye muscle area (EMA), carcass weight (CWT) and back fat thickness (BFT), using the standard 50 k array for the k-means and random selection cross-validation (CV). Vertical lines indicate the empirical standard error for each CV result

The accuracy of prediction of breeding values for carcass traits was, on average, 0.06 higher when using a random sampling strategy to perform the cross-validation compared to using a k-means clustering strategy (average accuracy is 0.58 vs 0.52; Fig. 1a). This is likely due to predicting animals in the validation set that are less related to the training set when selection is based on the k-means strategy. The difference between the sampling strategies was similar for all traits (ranging from 0.04 to 0.07). The bias was low (from − 0.02 to 0.005) when the random strategy was used for the sampling, while bias was higher with the k-means strategy (ranging from 0.05 to 0.13) (Fig. 1b).

#### Comparison of the accuracy of genomic predictions based on different dataset sizes

The accuracy of prediction for each size of the reference-validation dataset is in Table 3. For this comparison, we used GBLUP with one ${\mathbf{G}}$ matrix (${\mathbf{G}}_{1}$ in Model 2) and the 50 k SNP array. For all traits, with the larger reference-validation (RV) dataset (n = 12,717) accuracies were higher (by 0.02–0.04) than with the smaller RV dataset (n = 9717). These differences were not statistically significant but they were consistent across all traits. The reduction in the accuracy of prediction for the RV dataset with 10,717 animals was small and allowed us to use an independent discovery dataset of 3000 animals. Therefore, the RV dataset of 10,717 animals was used to compare different methods for the genomic prediction analysis.

**Table 3:** Accuracies of prediction of breeding value (empirical CV standard error in brackets) for carcass traits in Hanwoo cattle using different sizes of reference-validation (RV) datasets

Trait	Dataset size			
	RV = 12,717	RV = 11,717	RV = 10,717	RV = 9717
MS	0.50 (0.02)	0.47 (0.01)	0.47 (0.02)	0.46 (0.02)
EMA	0.53 (0.02)	0.51 (0.02)	0.50 (0.02)	0.50 (0.02)
CWT	0.59 (0.03)	0.56 (0.02)	0.58 (0.03)	0.57 (0.02)
BFT	0.47 (0.02)	0.46 (0.02)	0.47 (0.02)	0.43 (0.03)

*BFT* back fat thickness, *EMA* eye muscle area, *CWT* carcass weight, *MS* marbling score

#### Pre-selection of SNPs based on GWAS

**Fig. 2:**
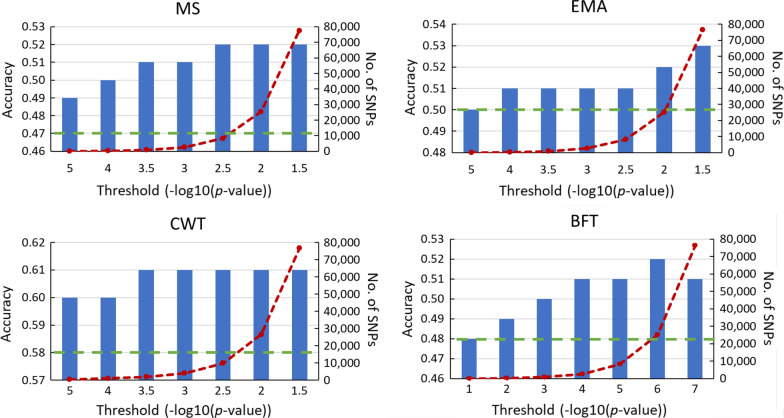
Accuracy of genomic prediction of breeding value (bars) for carcass traits marbling score (MS), eye muscle area (EMA), carcass weight (CWT) and back fat thickness (BFT) by using a 50 k standard SNP array and top SNPs from GWAS (3000 animals) added with various significance thresholds (red dashed line). The green dashed line indicates the accuracy of prediction from using a 50 k SNP array only. Results are based on cross-validation with 10,717 animals

To investigate what is the optimal number of pre-selected top SNPs for genomic prediction, we evaluated the increase in accuracy of prediction when a second ${\mathbf{G}}$ matrix $({\mathbf{G}}_{2} )$ based on the selected SNP set was included in a GBLUP model for each trait (Fig. 2). We compared seven significant threshold values for SNP selection, with the − log_10_(*p*-value) equal to 5, 4, 3.5, 3, 2.5, 2 and 1.5, and using the independent discovery set of 3000 animals. The number of significant SNPs increased rapidly with lower threshold values whereas accuracy tended to be highest when the largest SNP set was used. There was only a limited amount of overlap of the SNPs identified between the discovery datasets, and it was largest for CWT (see Additional file 1: Figure S1).

A threshold of 2.5 (− log_10_(0.0032)) resulted in the nearly highest improvement in accuracy of prediction for carcass traits although a moderate number of top SNPs (~ 8688) was used in ${\mathbf{G}}_{2}$ (Fig. 2). Therefore, this threshold was used to select the top SNPs from GWAS and to compare methods. Although an increase in accuracy was sometimes observed by including a larger number of SNPs (lower significant threshold) in ${\mathbf{G}}_{2}$ (Fig. 2; red dashed line), a more efficient use of the extra information might be achieved when a smaller number of pre-selected SNPs is included in SNP arrays.

Results from the GWAS based on imputed sequences revealed many strong signals across the genome for all traits with the most significant SNPs being found for CWT and BFT (results not shown). In general, the number of significant SNPs identified for CWT was largest (10,892) with ~ 8000 significant SNPs identified for the other traits (Fig. 2).

Based on the results from the largest discovery dataset (4000 animals), a list of the candidate genes that are located close to the most significant SNPs with a high threshold value of *p* < 1.0E−05 was identified (see Table 4). CWT is the trait with the largest number of genes identified, most of which are located on *Bos taurus* chromosome (BTA) 4, 10 and 14.

**Table 4:** Genes located close to significant SNPs (p < 1.0E−05) associated with carcass traits from GWAS on a discovery dataset of 4000 animals

Gene name (symbol)	Trait	SNP position (Chr:bp)	MAF	p-value	%
*ENSBTAG00000039810*	MS	23:33569238	0.31	6.13E^−06^	3
*N-ethylmaleimide-sensitive factor attachment protein, gamma* (*NAPG*)	EMA	24:42615958	0.32	8.29E^−06^	3
*ENSBTAG00000033237*	EMA	29:36927709	0.44	6.32E^−06^	3
*Epidermal growth factor receptor pathway substrate 15 like 1* (*EPS15L1*)	BFT	7:6484738	0.06	9.94E^−06^	2
*SAM* *pointed domain containing* *ETS* *transcription* *factor* (*SPDEF*)	BFT	23:8537156	0.18	7.04E^−06^	2
*HEPACAM family member 2* (*HEPACAM2*)	CWT	4:10371904	0.11	1.75E^−06^	3
*G protein subunit gamma transducin 1* (*GNGT1*)	CWT	4:11059866	0.10	4.66E^−08^	4
*ENSBTAG00000035660*	CWT	4:13589367	0.05	9.43E^−08^	4
*Fidgetin like 1* (*FIGNL1*)	CWT	4:5373926	0.10	3.15E^−07^	4
*ENSBTAG00000018039*	CWT	10:95431561	0.47	5.99E^−06^	3
*Coiled-coil-helix-coiled-coil-helix domain containing 7* (*CHCHD7*)	CWT	14:25059742	0.34	3.23E^−10^	6
*Ribosomal protein L39* (*RPL39*)	CWT	14:26181231	0.17	8.75E^−09^	5
*UBX domain protein 2B* (*UBXN2B*)	CWT	14:26303702	0.28	3.41E^−06^	3
*Thymocyte selection associated high mobility group box* (*TOX*)	CWT	14:26941314	0.18	1.08E^−06^	4
*ENSBTAG00000014045*	CWT	14:29678929	0.24	8.54E^−06^	3
*KDEL endoplasmic reticulum protein retention receptor 2* (*KDELR2*)	CWT	25:38887854	0.19	3.07E^−06^	3

*Chr* chromosome, *bp* base pairs, *MAF* minor allele frequency; *%* percentage of variance explained by the genotype

#### Pre-selection of SNPs based on gene expression

A large numbers of SNPs were pre-selected based on the results from gene expression (Table 5). A significance threshold of the *p*-value < 0.0032 was set to include as many eQTL as possible since the SNPs in high LD were removed and because considering a stringent *p*-value could leave out important information. In total, 452,258 unique eQTL were detected across all ages (Table 5). We identified 76,018, 45,779, 38,742, 58,890, 354,530 eQTL at 8, 12, 18, 24, and 30 months of age, respectively, with on average 24,448 eQTL shared across all ages. After combining the lists of the eQTL for all ages and removing the SNPs in high LD (r^2^ > 0.95), 130,748 unique SNPs remained. The exact number of SNPs per reference-validation dataset is in Table 5. The number of SNPs that were pre-selected from the GSA results ranged from 19,000 to 36,000 SNPs after removing SNPs in high LD (r^2^ > 0.95), depending on the trait (Table 5).

**Table 5:** Number of SNPs used in the genomic prediction, pre-selected from gene expression analysis and after pruning (and the SNPs remaining in the 50 k -G50adj in brackets) in the various RV subsets

	Genes	SNPs	Reference-validation datasets			
			12,717	11,717	10,717	9717
eQTL	10,224	452,258	130,750 (40,129)	130,251 (40,135)	130,080 (40,130)	130,582 (40,124)
GSA_MS_	473	98,099	23,398 (40,774)	23,338 (40,776)	23,280 (40,778)	23,366 (40,769)
GSA_EMA_	367	76,585	19,852 (40,805)	19,799 (40,803)	19,759 (40,804)	19,817 (40,806)
GSA_CWT_	440	104,512	24,361 (40,740)	24,298 (40,743)	24,268 (40,744)	24,327 (40,739)
GSA_BFT_	810	146,958	36,219 (40,661)	36,116 (40,658)	36,056 (40,633)	36,168 (40,658)

*GSA* gene expression significantly associated, *BFT* back fat thickness, *EMA* eye muscle area, *CWT* carcass weight, and *MS* marbling score

#### Comparison of prediction methods

**Fig. 3:**
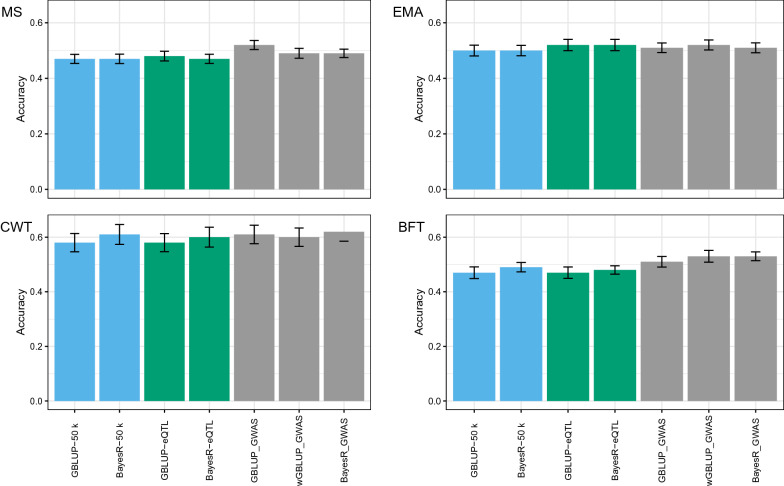
Accuracy of genomic prediction of breeding value for carcass traits marbling score (MS), eye muscle area (EMA), carcass weight (CWT) and back fat thickness (BFT) comparing GBLUP, wGBLUP, and BayesR models using three SNP sets: (1) standard 50 k array (blue bars); (2) 50 k and eQTL-SNPs (green bars) and (3) 50 k and GWAS top-SNPs (grey bars). All analysis use cross-validation (CV) with 10,717 animals. Vertical lines indicate the empirical standard error for each CV result

We compared different methods for genomic prediction using 10,717 animals as RV dataset (Fig. 3). Regardless of the method of preselecting SNPs, we observed no large differences between the methods evaluated. Comparing the achieved accuracies from adding pre-selected SNPs, the top SNPs from GWAS were the best source of information to improve the accuracy of prediction for carcass traits. For all traits, there were small improvements in the accuracies of prediction when using pre-selected SNPs from the eQTL (Fig. 3) but no benefit was observed from using SNPs from GSA (not shown).

Results from wGBLUP and GBLUP were compared for both Models 2 and 3 (using either 50 k or 50 k and top-SNPs from the GWAS discovery set of 3000 animals (50 k_GWAS; Table 6). Accuracies of prediction were similar regardless of the number of iteration rounds used, although accuracy of prediction tended to be higher after two or three iteration rounds. When using the 50 k panel, wGBLUP showed a small improvement in accuracy over the standard GBLUP method of 0.01 for BFT, whereas for MS and EMA the accuracy was not improved. The use of the 50 k_GWAS SNP panel in wGBLUP improved the accuracy of prediction slightly more, i.e. from 0.01 to 0.05 (Table 6).

**Table 6:** Accuracy of prediction of breeding value for carcass traits (SE) in consecutive iterations of wGBLUP with 50 k and a combination of 50 k and top-SNPs from GWAS (50 k_GWAS)

Model	Iteration	MS	EMA	CWT	BFT
50 k (GBLUP)	1	0.47 (0.02)	0.51 (0.02)	0.58 (0.03)	0.48 (0.02)
50 k (wGBLUP)	2	0.47 (0.02)	0.51 (0.02)	0.58 (0.03)	0.48 (0.02)
	3	0.47 (0.02)	0.50 (0.02)	0.59 (0.03)	0.49 (0.02)
	4	0.47 (0.02)	0.49 (0.02)	0.58 (0.03)	0.50 (0.02)
	5	0.46 (0.02)	0.48 (0.02)	0.58 (0.03)	0.49 (0.02)
50 k_GWAS (wGBLUP)	2	0.49 (0.02)	0.52 (0.02)	0.60 (0.03)	0.52 (0.02)
	3	0.49 (0.02)	0.51 (0.02)	0.60 (0.03)	0.53 (0.02)
	4	0.49 (0.02)	0.51 (0.02)	0.60 (0.03)	0.53 (0.02)
	5	0.48 (0.02)	0.50 (0.02)	0.59 (0.03)	0.52 (0.02)

*BFT* back fat thickness, *EMA* eye muscle area, *CWT* carcass weight, and *MS* marbling score

For MS, the use of top SNPs increased the accuracy of prediction by 0.05 for GBLUP-GWAS, and by 0.02 for wGBLUP-GWAS and BayesR-GWAS (Fig. 3). Pre-selected SNPs from eQTL and GWAS improved the prediction for EMA in all models compared with the 50 k baseline (Fig. 3). The largest improvement in prediction for CWT was achieved with BayesR-GWAS and GBLUP-GWAS by 0.04 and 0.03, respectively. For BFT, the largest improvement was observed when the wGBLUP-GWAS and BayesR-GWAS models were implemented with an increase of 0.06 (Fig. 3). For MS, BFT, and CWT, the addition of SNPs from GWAS increased the accuracy of prediction compared to using SNPs from eQTL whereas using eQTL SNPs proved to be the same or better than the GWAS SNPs for the prediction of EMA.

We found no accuracy increase for CWT and BFT when comparing results from using ${\mathbf{G}}_{1}$ based on the 50 k standard array and using a second ${\mathbf{G}}_{2}$ from SNPs that were selected based on eQTL, but accuracies of prediction for EMA improved by 0.02 (Fig. 3).

**Fig. 4:**
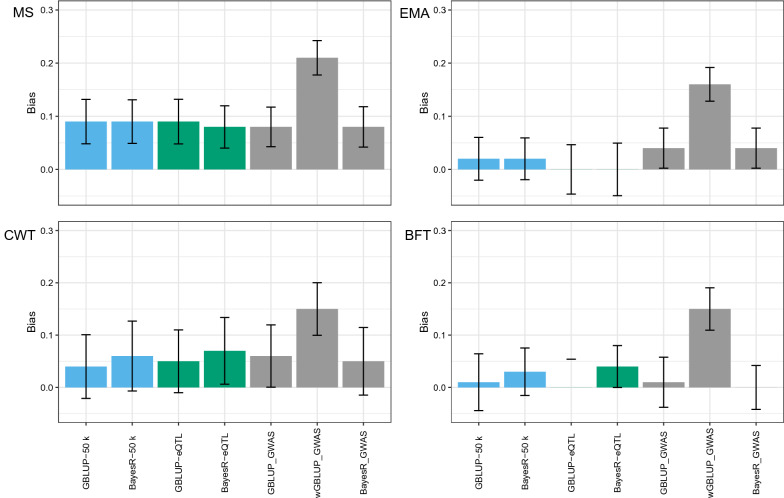
Bias of genomic prediction of breeding value for carcass traits marbling score (MS), eye muscle area (EMA), carcass weight (CWT) and back fat thickness (BFT) comparing GBLUP, wGBLUP, and BayesR models using three SNP sets: (1) standard 50 k array (blue bars); (2) 50 k and eQTL-SNPs (green bars) and (3) 50 k and GWAS top-SNPs (grey bars). All analyses use cross-validation (CV) with 10,717 animals. Vertical lines indicate the empirical standard error for each CV result

Genomic predictions for all carcass traits showed some bias but the bias observed with wGBLUP was generally much higher than that with the other methods (Fig. 4). The biases observed for EMA and BFT were lower than for MS and CWT (Fig. 4).

## Discussion

The aim of this study was to assess the accuracy of prediction for carcass traits in Hanwoo cattle when using GBLUP, wGBLUP, and a BayesR model, and the potential gain from using pre-selected SNPs from a GWAS on imputed sequence data as well as from gene expression information. We found that pre-selected SNPs from GWAS improved the accuracy of prediction slightly, whereas gene expression SNPs that are located in genes, the expression of which is associated with phenotypic differences in carcass traits, were less useful to improve prediction accuracy.

During the last decade, GBLUP was applied in many breeding programs for plants and animals and resulted in increased rates of genetic gain [40, 41]. To date, genomic selection is not commonly applied in Hanwoo cattle breeding programs to improve carcass traits. In this study, first we established a baseline accuracy of prediction for Hanwoo cattle, using the standard 50 k SNP array and a ten-fold cross validation, and compared the accuracy obtained from k-means cross-validation with random selection. Higher accuracies (an increase ranging from 0.04 to 0.07, depending on the trait) were observed with random CV. Since the k-means CV strategy is based on clustering according to the relatedness between samples, it is less likely that animals in the validation set have related animals in the reference training set. Therefore, relatedness between training and test animals has a fairly large effect on accuracy of prediction, as was previously pointed out in the literature [42]. We also observed that, with a random CV strategy, the benefit of using top-SNPs was smaller (results not shown). The benefit of including prioritized SNPs for genomic prediction is larger when the genomic relationships between the reference and validation sets are lower [17]. Since the predictive SNPs were selected based on an association with the phenotype in relatively unrelated individuals, their contribution to accuracy of prediction should be less affected by relatedness. Similarly, we reported an improvement in accuracy of prediction when a larger reference population was used, as previously reported [43, 44], which also reduces the marginal value of using more predictive SNPs. Thus, the benefit of using more predictive SNPs is most likely greater when the baseline accuracy is lower and when the individuals being predicted have a lower relatedness to the reference population.

To select the GWAS discovery dataset, the more genetically diverse animals were chosen, resulting in a lower degree of LD in the discovery set and therefore a higher resolution of the QTL regions identified. Previous studies have shown that SNPs that are located near QTL detected in multibreed datasets tend to be more useful in improving accuracy of prediction [8, 45], and this is expected to be due to the lower LD that exists in multibreed datasets. In such a case, detection of QTL would require a higher marker density, but fewer markers would be in LD with the QTL across a set of more diverse individuals, hence allowing more precise mapping of the QTL. While we did not use a multi-breed discovery set, a greater diversity in the discovery set of a single breed might also prove to be more useful.

The use of more individuals for the GWAS is expected to improve the identification of associated SNPs. The use of imputed sequence genotype data increased the number of detected QTL and their *p*-values compared with 50 k (results not shown) especially for traits that seem to behave more highly polygenic (MS, EMA, and BFT). However, a larger number of variants might also lead to more false positives. The amount of overlap in QTL between the various discovery sets was small in our study, which suggests that most of the significant SNPs might not be real QTL and could partly explain the limited increase in accuracy from using the top SNPs. Although the size of the dataset used in this study was relatively large, at least for beef cattle, it is clear that even larger datasets are needed in GWAS to successfully identify SNPs that will increase accuracy of prediction.

There is no consensus in the literature about the threshold that should be used for selecting top SNPs for the purpose of improving prediction. In this study, we tried to keep a balance between the number of SNPs used and the increase in the accuracy of prediction. In general, the use of larger discovery datasets improved the identification of informative SNPs and these SNPs did increase the accuracy of prediction for all the traits. The top-SNPs that increased the accuracy of prediction were identified using a low significance threshold (*p*-value < 0.0032), and after filtering for LD (r^2^ > 0.95), less than 30% of the SNPs were kept (Table 5).

The use of the larger dataset for GWAS did allow to identify QTL regions associated with carcass traits located on the *NAPG*, *EPS15L1*, *SPDEF*, *HEPACAM2*, *GNGT1*, *FIGNL1*, *CHCHD7*, *RPL39*, *UBXN2B*, *TOX*, and *KDELR2* genes among other non-annotated genes (Table 4). In a previous study in Hanwoo cattle, BTA4, 6, and 14 were significantly associated with CWT and EMA [14]. The *EPS15L1* gene is an essential component of the endocytic pathway [46]. The *SPDEF* gene is known to be associated with BFT in pigs [47]. The *HEPACAM2* gene was previously identified in a QTL region for mid-test metabolic weight (MMWT) in SimAngus on BTA4 [48]. The *CHCHD7* gene on BTA14 is associated with CWT in the Hanwoo breed [11], with knuckle, biceps and shank trait in Chinese Simmental cattle [49], with carcass weight in Japanese Black steers [50], with back fat thickness and rump fat thickness in the Nellore breed [51], and with fat thickness and intramuscular fat in composite cattle [52]. The *UBXN2B* gene is associated with MMWT in SimAngus [48], and with carcass weight, carcass fat, and carcass conformation in Simmental [53]. The *TOX* gene is located within the QTL that is associated with CWT and EMA in Hanwoo cattle [14], and with residual feed intake and MMWT [48]. The expression of *KDELR2* is upregulated in response to endoplasmic reticulum stress to stabilize exocytosis [54] but no studies have reported an association of this gene with CWT in cattle.

Although our GWAS results revealed some SNPs that are located on well-known genes for each trait, especially for CWT (Table 4), larger datasets are needed to refine the regions that are associated with EMA, MS, and BFT. We observed that, after pruning, the SNPs were not always located close to the polymorphisms within informative functional genes that help to understand the biological nature of the traits. Nevertheless, overall these markers explain some of the genetic variation associated with the traits in this study and had a small to modest effect on the accuracy of predicting GBV. Hanwoo cattle have a small effective population size, which has an increasing effect on the LD between SNPs and causal variants, but this argument holds for SNPs in the standard 50 k array as well as for SNPs selected from the sequence data. Further evaluation of optimal ways of selecting SNPs from a discovery population and SNP filtering methods based on LD and allele frequency are recommended to optimize the identification of the most suitable QTL for improving genomic selection.

Applying an additional ${\mathbf{G}}$ matrix for the top SNPs in the GBLUP model effectively gives more weight to the top-SNPs, since few SNPs share a relatively large variance component to calculate the GBV. Our results show that this model is more effective to improve the accuracies for all carcass traits in Hanwoo cattle (Fig. 4), even for polygenic traits such as MS and EMA. The observed improvement in accuracy of prediction suggests that these pre-selected SNPs indeed contain some additional information compared to neutral markers on the standard 50 k SNP array. Brøndum et al. [55] reported that the use of top-SNPs together with the 50 k SNP chip in a GBLUP model increased the accuracy of prediction by 5% for production traits in dairy cattle. In a study based on sheep data, the use of two ${\mathbf{G}}$ matrices (50 k and significant variants from the GWAS) to estimate GBV led to an increase in accuracy of prediction by on average 6.2 and 4.1% for purebred and crossbred sheep, respectively [8].

In previous studies, the advantage of BayesR over GBLUP was more pronounced for traits that are influenced by few QTL with moderate to large effects. The BayesR method resulted in a higher accuracy (on average 0.05 across traits) than GBLUP for milk yield and fat yield but there was no difference in the accuracy of prediction for protein yield [56]. Similarly, our results for CWT and BFT, for which more significant QTL regions were found, show that the use of BayesR-50 k increased the prediction accuracy by 0.03 and 0.02 compared to that of GBLUP-50 k (Fig. 3), whereas there was no improvement for the other traits. In addition, a larger improvement in accuracy of prediction was observed for BFT (0.06) and CWT (0.04) when BayesR with pre-selected SNPs (BayesR-GWAS) was used. In sheep, the use of top SNPs in combination with BayesR increased the accuracy by 0.09 in Merino and 0.06 in crossbreds [8].

The use of top SNPs in wGBLUP also improved the accuracy but only by 0.02 for MS, 0.01 for EMA, 0.02 for CWT, and 0.06 for BFT (Fig. 3). However, the wGBLUP-GWAS method also tended to increase the bias of the prediction quite severely, by 0.11 to 0.14, depending on the trait (Fig. 4). After reaching the fifth iteration, a higher bias was observed. Similarly, Lopez et al. [37] reported a reduction in the regression coefficient (increased bias) at each additional iteration. This increase was expected since we assigned more weight to SNPs with a large effect in the training population but these SNP effects tended to be overestimated. An increase in bias was also reported by [57] in a single-step wGBLUP strategy.

The differences observed in the performance of the methods depend strongly on the genetic structure of each trait. For polygenic traits such as MS, we observed that the performance of GBLUP-GWAS was superior to that of Bayes-GWAS. In a simulated dataset, the use of BayesR for highly polygenic traits resulted in more variable heritability estimates and slightly lower accuracies of prediction compared to GBLUP [39]. A simulation study testing the wGBLUP method showed that a trait with a larger number of QTL has a smaller increase in accuracy (0.05) than a trait with an only small number of QTL whereas the accuracy could increase by 0.1 when the weighting SNPs effects were considered in the wGBLUP model [58].

In our study, we used imputed SNPs at the sequence level and these imputations were derived from other imputed sequence data. Imputed sequence data could potentially include errors and these errors would be further propagated when the data are used as a reference for further imputation of other samples. We used whole-genome sequence SNPs that had been imputed by a Minimac3 accuracy higher than 0.4, but the accuracy of imputation might be inflated if imputed genotypes were used as a reference. It has been reported that imputation errors [59] and especially errors in the imputation to whole-genome sequences data could lower the accuracy of prediction by 0.01 to 0.03 [3]. Moreover, accuracy of imputation tends to be lower for SNPs with a lower MAF [60]. The use of larger and more diverse reference populations as well as a larger sample of individuals in the reference set with real sequence data should be considered for imputation purposes to better capture rare markers [61].

For complex traits, the use of more biological information (protein, metabolite, etc.) has been suggested to identify genomic features that are enriched for causal variants, and these were shown to increase the accuracy of prediction slightly by using the SNPs located in the best-performing gene ontology terms information [18]. In our study, no improvement was observed in the accuracy of prediction of any trait using the SNPs that were selected from GSA, although a significantly larger number of markers were included in the ${\mathbf{G}}_{2}$ (from ~ 19,000 to 36,000 SNPs) compared to using only the 50 k SNP panel. It is possible that SNPs located in these genes have a low level of heterozygosity and therefore only a small part of the variance of the phenotype is explained. It is also important to point out that only 45 animals were used in the gene expression study and this is a limited number of samples for identifying the GSA genes. Moreover, these gene expression patterns might change with tissue and development stage of sampling. The expression profile (GSA) from the *longissimus dorsi* muscle may have a small effect on traits, such as CWT and BFT. In another study, the use of SNPs from a high-density panel (800 k) that were located in exons did not improve the accuracy of prediction over the 50 k or 800 k SNP chips when analyzing milk traits in a model that included these SNPs [56].

Simulation studies have shown that using the actual causal variants could significantly increase the accuracy of prediction [62], but this increase does depend on the size of the QTL detected [42]. Wang et al. [63] reported that the use of true QTL in combination with non-QTL-markers decreased the accuracy of prediction. Therefore, to what extent are the selected SNPs actual causal variants, or at least in very high LD with causal variants is important for their usefulness for prediction. GWAS studies based on sequence data are often still underpowered, given the large multiple testing challenge, but at least larger GWAS datasets might be able to better select the SNPs that can explain and predict genetic variation. While expression studies can help find functional genes relevant to phenotypes of interest, fundamentally they do not detect SNPs based on their ability to explain variation in those phenotypes. Moreover, these studies are usually much smaller in size and depend largely on the timing and type of tissue sampled. Therefore, expression studies might be useful to support GWAS to detect more predictive SNPs, but when used alone, they have a lower chance of being successful than GWAS studies for that purpose.

## Conclusions

For all traits, the accuracy of prediction slightly improved by pre-selecting SNPs from GWAS, whereas only a slight and non-significant improvement was obtained when using eQTL SNPs. The use of SNPs selected from GSA did not lead to an improvement in the genomic prediction of carcass traits compared to the standard 50 k SNP panel. The performance of each method for estimating GBV is trait-specific and likely depends on the genetic architecture of each trait. The GBLUP-GWAS method reached a higher accuracy of prediction for MS, whereas the BayesR method was better for CWT and BFT. In future studies, the use of a larger discovery dataset for GWAS will help to improve the identification of more QTL regions and therefore will potentially increase the accuracy of prediction for carcass traits especially for traits, such as EMA and MS. In addition, larger and more targeted gene expression studies should be used and combined with GWAS to show whether these studies have potential to provide more predictive SNPs.

## Supplementary information

**Additional file 1: Figure S1.** Venn diagram with the number of samples that overlap between four sizes of the discovery dataset (D; from 1000 to 4000 animals). Marbling score (MS), eye muscle area (EMA), carcass weight (CWT), and back fat thickness (BFT).

## Data Availability

Not applicable.
