# Supplementary material for: Use of gene expression and whole-genome sequence information to improve the accuracy of genomic prediction for carcass traits in Hanwoo cattle
Source: Genet Sel Evol. 2020 Sep 29;52:54. doi: 10.1186/s12711-020-00574-2 (PMC7525992; doi:10.1186/s12711-020-00574-2)
Supplement: Supplementary file 1 — Additional file 1: Figure S1. Venn diagram with the number of samples that overlap between four sizes of the discovery dataset (D; from 1000 to 4000 animals). Marbling score (MS), eye muscle area (EMA), carcass weight (CWT), and back fat thickness (BFT). [file 12711_2020_574_MOESM1_ESM.docx]

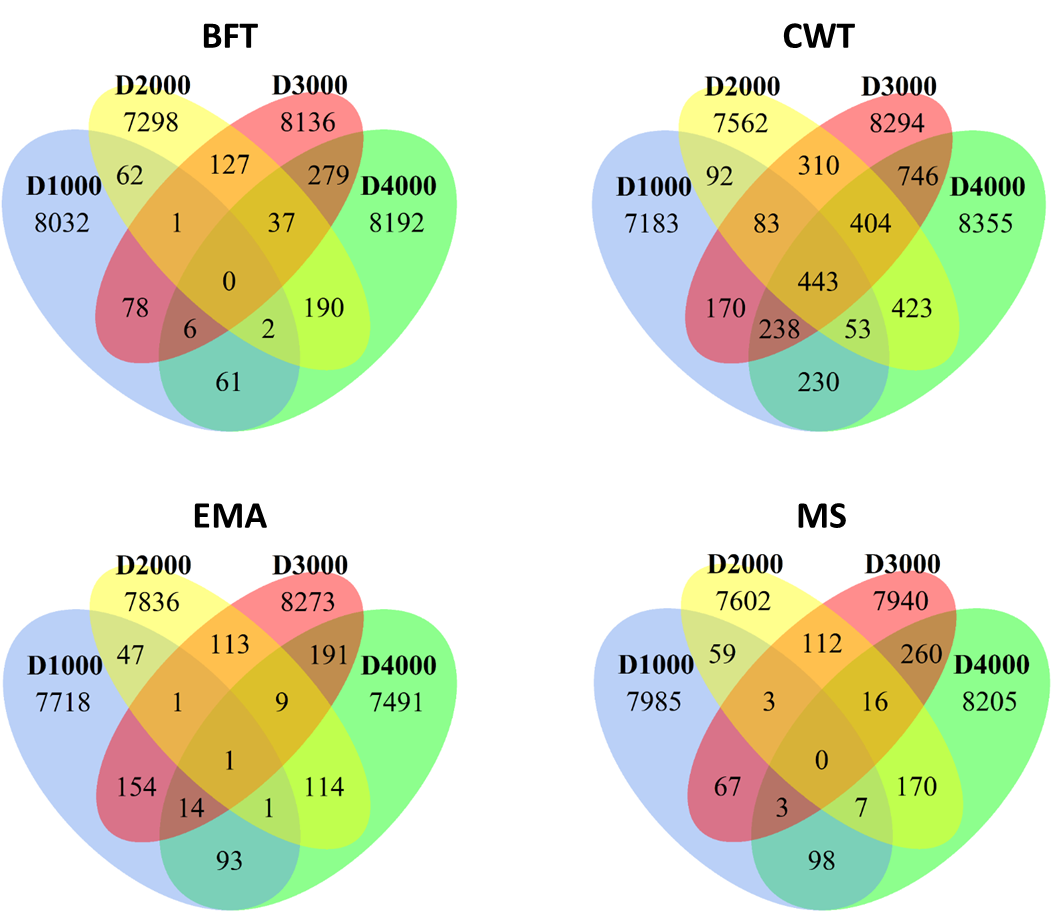


**Figure S1.** Venn diagram with the number of samples overlapping between four different sizes of discovery datasets (D; from 1,000 to 4,000 animals). Marbling score (MS), eye muscle area (EMA), carcass weight (CWT), and back fat thickness (BFT).
